# Surface Sensitivity of Mesoporous Carbon-Supported Iron Catalysts in the Fischer–Tropsch Synthesis: An *In Situ* XPS Site Evolution at Distinct Reaction Conditions

**DOI:** 10.1021/acs.energyfuels.5c02269

**Published:** 2025-08-07

**Authors:** João Pedro S. Nascimento, Alcineia C. Oliveira, Fabiano A.N. Fernandes, Gardênia S Pinheiro, Juan Pedro Holgado, Alfonso Caballero Martinez, Olga Guerrero-Pérez, José Jiménez-Jiménez, E. Rodríguez-Castellón

**Affiliations:** 1 Departamento de Química Analítica e Físico-Química, 28121 Universidade Federal do Ceará, Campus do Pici-Bloco 940, Fortaleza, Ceará 60355-636, Brazil; 2 Departamento de Engenharia Química, 28121 Universidade Federal do Ceará, Campus do Pici-Bloco 709, Fortaleza, Ceará 60440 900, Brazil; 3 Universidade Federal do Piauí, Campus Universitário Ministro Petrônio Portella - Bairro Ininga, Teresina, Piauí 64049-550, Brazil; 4 Instituto de Ciencia de Materiales de Sevilla, 16778 Universidad de Sevilla, Av. Americo Vespucio s/n, Sevilla 41092, Spain; 5 Departamento de Química Inorgánica, Facultad de Ciências, 16752 Universidad de Málaga, Málaga 29071, Spain

## Abstract

The Fischer–Tropsch process can be considered as an alternative route to convert fossil fuels such as crude oil, coal, and methane for the production of more environmentally friendly liquid fuels and chemicals. As recoverable sources of carbon, Fischer–Tropsch synthesis (FTS) converts a mixture of CO and H_2_ to a range of hydrocarbons, which is free of sulfur and nitrogen and low in aromatics. The surface-sensitive investigation of the temperature and pressure effects on the FT synthesis performance over mesoporous carbon-supported iron catalysts was examined by *in situ* X-ray photoelectron spectroscopy analyses. Raman and Mössbauer spectroscopy measurements illustrated the structural properties of mesoporous Fe-based oxides. Under FTS reaction conditions of 20 and 30 atm, and temperatures of 240, 255, and 270 °C with a CO-to- H_2_ ratio of 1, the solids were active with 38–45% of CO conversion and a rate of 1 × 10^–5^ mol_CO_·g^–1^·s^–1^. The product distribution gave C_1_–C_4_, C_5_–C_9_, and C_10_^+^ products with the structure of the solid marginally affected by the type of product obtained. The *in situ* surface XPS analyses were conducted at ∼240–270 °C and 10 atm with a CO-to-H_2_ ratio of 1 for 1 h. The α-Fe_2_O_3_ phase was reduced to Fe_3_O_4_ resulting in well-dispersed magnetite nanoparticles with further reduction to metallic iron on the mesoporous carbon support. Such α-Fe phase demonstrated accessibility of the syngas resulting in the activity of the solids. The chemical evolution of the Fe 2*p*, O 1*s*, C 1*s*, and K 2*s* core levels during the FTS with increasing the temperature up to 255 °C suggested that the surface carburization formed χ-Fe_5_C_2_ and θ-Fe_3_C iron carbide phases along with Fe_3_O_4_ and, tentatively, the metallic iron phase. The mesoporous carbon-supported iron catalysts having χ-Fe_5_C_2_ carbide determined the activity and stability during the FTS synthesis.

## Graphical abstract

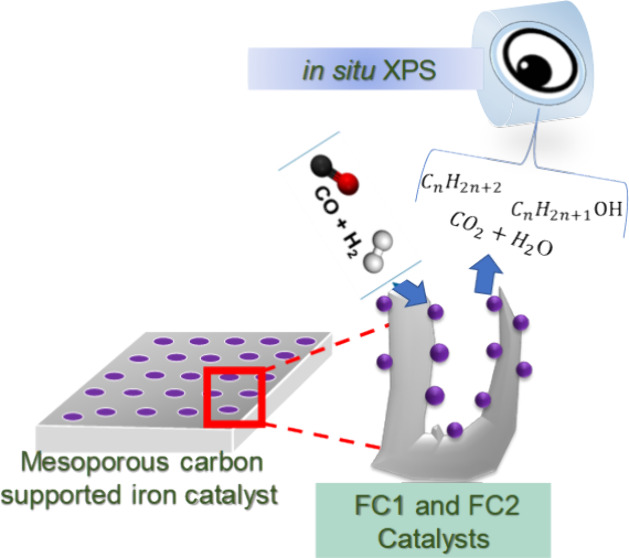

## 1 Introduction

Fischer–Tropsch synthesis (FTS) is a widely spread gas-to-liquid process in refining to convert synthesis gas, a mixture of CO and H_2_, into chemical feedstocks with high added values with *n* > 1 (eqs 1–3):^1–3^

$$\left(2n+1\right)H_{2}+n\mathrm{CO}\to C_{n}H_{2n+2}+nH_{2}O$$ 1

$$\left(2n\right)H_{2}+n\mathrm{CO}\to C_{n}H_{2n}+nH_{2}O$$ 2

$$\left(2n\right)H_{2}+n\mathrm{CO}\to C_{n}H_{2n+1}\mathrm{OH}+\left(n-1\right)H_{2}O$$ 3

$$H_{2}O+\mathrm{CO}↔{\mathrm{CO}}_{2}+H_{2}O$$ 4

The FTS is considered as an alternative route to transform noncrude feedstocks and fossil fuel including crude oil, coal, and methane into environmentally friendly liquid fuels and chemicals. As a recoverable source of carbon, the syngas is converted into a range of hydrocarbons, which is free of sulfur and nitrogen and low in aromatics.^2,3^

In this regard, FTS is a large-scale route industrially used to produce a range of synthetic liquid fuels and fine chemicals besides oxygenated compounds and waxes.^3–6^ The water gas shift reaction (WGS) in eq 4 constitutes a reversible parallel-consecutive reaction for converting CO to CO_2_ and water that can be controlled, when adjusting the initial H_2_/CO ratios.^3^

A number of catalysts have proven to be effective in FTS, mainly the iron- and cobalt-based oxides, since the past FTS commercial routes.^1,7–9^ Cobalt is one of the most actives frequently encountered for low-temperature FTS owing to its chemical stability, high selectivity for long-chain hydrocarbons, and low WGS reactivity.^1,8,10^ However, the particle's tendency to agglomerate disadvantage results in Co-based particles sintering with the consequent loss of stability and selectivity, in addition to low CO conversions.^10,11^ Iron-based catalysts are the most widely used, from the industrial standpoint, because of their lower cost, relatively high activity, and stability toward deactivation.^12,13^

Numerous attempts have been made to engineer new formulations for FTS iron-based catalysts comprising massic, supported, and promoted solids prepared under a variety of methods.^12–14^ Particularly, the catalytic activity of these iron-based oxides correlates with their most interesting characteristics such as stable structures, redox properties, tunable compositions, versatile surface chemistry, porosity, and nanostructured features, in some cases.^9,14^ All efforts, including synthesizing iron-based multifunctional materials for FTS, largely failed due to the shortcomings associated with phase transformation, sintering of Fe particles, and carbon deposition that should limit access to the surface active site inherently deactivating the solids over long periods of times and operation conditions.^14–16^

Therefore, improvement of the Fe-based catalysts for the FTS process turns out to be mandatory. Despite the considerable progress in the achievements of α,γ-Fe_2_O_3_-based catalysts and their similar-shaped structure reduction to obtain Fe_3_O_4_ and its further reduction to metallic iron, the carburization to form active iron carbide phases continues to be extensively studied in FTS reaction.^3,9,15^ To shed light on the nature of the iron carbide formation, the carburization occurs during FTS reaction steps on a complex mixture of iron carbides possessing a variety of carbon contents, i.e., Hägg carbide χ-Fe_5_C_2_, Fe_7_C_3_, cementite θ-Fe_3_C, ε-Fe_2_C, and ε′-Fe_2.2_C.^17,18^ This has attracted a great deal of research interest due to these iron carbides' potentially debatable activity and selectivities to liquid fuels and olefins.^17^

Nevertheless, under catalytic operating conditions, it can be assumed that the essential aspects of the dynamic FTS reaction mechanism involving the iron carbide structures are still limited. To date, the surface polymerization of CH_*x*_ monomer reaction mechanism over Fe-based catalysts prevails in FTS, when considering the carbon chain growth provided by iron carbide phases.^9,17,19^ Also, limitations such as a wide range of temperature, pressure gradients, and gas feed ratio effects based on direct experiments seem to play a major drawback to understand the carburization process with Fe-based oxide catalysts, owing to the heterogeneity of Fe particle sizes.^17,20,21^

The abovementioned assumptions pass through a challenge for experimental studies, and thus, spectroscopic investigations are currently used for assessing the structural and surface features of heterogeneous catalysts to establish structure–performance relationships.^15,20–22^ In this regard, the elucidation of deactivation of the solids and reaction mechanistic insights by a combination of spectroscopic techniques allow the design of catalysts to specific needs. This ultimately affords the iron-based oxide catalytic systems possessing distinct particle sizes and the redox properties of confined active iron species to be good candidates for controlling reactant and product distribution and selectivity in FTS.^17,19^

Moreover, the interaction of the Fe oxides with the gas reactants and products involved in the FTS reaction can dramatically affect their chemical composition and consequently their catalytic properties.^20^ For this reason, studies on the catalysts under working conditions using *in situ* and operando spectroscopy techniques are innovative approaches to understanding the reactivity and the role of the active phase in FTS.^19–21^ Given the fact of successful synthesis of Fe-based catalysts supported on polystyrene mesoporous carbons, the surface properties of these materials are of great interest toward the production of valuable liquid fuels and chemical feedstocks via FTS.^3^

Herein, the surface sensitivity of mesoporous carbon-supported Fe oxide catalysts is investigated under realistic conditions by *in situ* XPS spectroscopic investigations. Unlike *ex situ* techniques, the catalytic *in situ* XPS methodology for FTS reaction has not been developed well and few reports exist.^19,20^

The novelty of this work consists of understanding the transformation of the Fe oxide catalyst structures and the effects of the experimental FTS reaction conditions on the chemical evolution of the formed carbide phases. Thus, structure–activity relationships of the two distinct formulations are examined by Raman and Mössbauer spectroscopy measurements combined to *in situ* XPS analyses.

## 2 Experimental Section

### 2.1 Catalyst Preparation

The mesoporous carbon-supported iron oxides were prepared by a wetness impregnation method. A detailed description of the catalyst preparation has been presented in our previous reports.^3,8^ Briefly, the carrier consisting of a polystyrene-based spherical mesoporous carbon was purchased from Blucher GmbH Erkrath Germany as a fine powder and used without further modifications. Typically, a ferric nitrate in aqueous solution was impregnated onto 2.0 g of polymeric carbon support (C) in a rotary evaporator at 60 °C. The powdered solid consisting of 10 wt % Fe was dried in an oven at 60 °C and subsequently heated under flowing nitrogen at 500 °C for 2 h. The catalyst was designed as FC1. Another solid was prepared by using a similar procedure with the resultant catalyst calcined at 700 °C under the abovementioned conditions. The sample was denoted as FC2.

### 2.2 Characterizations

A summary of the fresh sample characterizations by X-ray diffraction (XRD), nitrogen physisorption isotherms, scanning electron microscopy coupled to energy-dispersive spectroscopy (SEM-EDS), and thermo-programmed reduction (TPR) are given in in our previous work.^3^

The chemical compositions and valences of elements in the spent solids were characterized by X-ray photoelectron spectroscopy (XPS). The analyses were carried out in a Physical Electronics VersaProbe II Scanning XPS photoelectron under an ultrahigh vacuum. A monochromatic X-ray Al Kα beam source was used. The binding energies were calibrated with respect to that of the C 1*s* peak at 284.8 eV for adventitious carbon. The spectra were analyzed using XPS software, and the high-resolution spectra were deconvoluted based on the Gaussian–Lorentzian curves using Origin 8.5 software.

Raman spectroscopy measurements were obtained to examine the vibrational properties of the spent samples. The Raman spectra were collected in a Jobin Yvon from a Horiba T64000 spectrometer. The Raman system was operated using an exciting radiation of 514 nm with a He–Ne laser. The spectrometer was coupled to a nitrogen-cooled charge-coupled-device detector. The scattering bands were calibrated with a reference peak at 520 cm^–1^ from a silicon wafer. A laser power below 15 mW for the sample was used to prevent sample degradation. Raman spectra were recorded using a 50× microscope objective lens. The spectra were collected with five data acquisitions per 10 s in the range 100–1600 cm^–1^ with a spectral resolution of 4 cm^–1^ and a wavenumber precision of 1 cm^– 1^. The Raman spectra were acquired at several locations on each sample to verify the homogeneity of the surface atoms in the solids.

Mössbauer spectra were collected on a WissEl spectrometer with transmission geometry. Samples were obtained at room temperature using a ^57^Co-in-Rh matrix source at 50 mCi. The calibrations were performed against a 6 μm-thick α-Fe foil. Spectra were fitted by using a least-squares nonlinear computer fitting program with constraints. Peaks were extracted by fitting the spectrum with Lorentzian functions possessing equal widths for each spectrum component. The baseline was fitted to a second-order polynomial, and the velocity nonlinearity was fitted to a third-order polynomial.

### 2.3 Catalytic Evaluation in the FTS Synthesis

Fischer–Tropsch synthesis was conducted using a semibatch high-pressure autoclave reactor from Parr Instruments model 4571. About 2 g of the solids were suspended in 200 mL of squalene under constant stirring of 800 rpm. Prior to the catalytic runs, the catalyst was activated *in situ* at 240 °C and 20 atm of pressure for 6 h. The catalytic runs were performed, feeding H_2_ and CO with a molar ratio of 1 in a temperature range of 240–270 °C and pressure of 20 or 30 atm.^8,12^ The catalytic runs were performed in triplicate. The products were analyzed in a gas chromatograph (Model Thermos Ultra), equipped with thermal conductivity (TCD) and flame ionization (FID) detectors. The calculated carbon mass balances were higher than 95%.

### 2.4 *In situ* XPS Evaluation of FTS Reaction

*In situ* surface XPS analyses were carried out at 10 atm in the temperature range of 240–270 °C, using a CO-to-H_2_ ratio of 1. Indeed, these experiments were conducted to simulate the catalytic environment to understand the evolution of the catalyst surface and its structure and correlate with the FTS. XPS analyses were performed with a high-temperature–high-pressure cell (SPECS HPC 20) with an infrared sample heating instrument. The apparatus allows a fast postreaction solid transfer from the reaction chamber to the spectrometer chamber to ensure the UHV conditions in the absence of laboratory air. A non-monochromatic Al Kα source of 1486.6 eV was used for spectral acquisition in a hemispherical analyzer (SPECS PHOIBOS 100) with 20 mA and 12 kV, which worked at fixed transmission and 50 eV pass energy with an energy step of 0.1 eV. Binding energies were calibrated with the reference of Al 2*p* at 74.0 eV. Such a methodology of calibration was applied due to the surface charging that may occur under photoemission conditions. Therefore, the effect was constant during spectral analyses and lower than 5 eV to avoid peak shape distortions. Before the analyses, samples were evacuated under a vacuum of 10^–7^ mbar at room temperature. Then, the solids were pretreated using a gaseous flow mixture of 50% hydrogen and argon for 2h at 1 atm. Subsequently, the samples were cooled to room temperature and transferred to the spectrometer chamber for XPS analysis. Afterward, the experimental setup consisted of placing the samples in the spectrometer chamber and subsequently to the high-pressure cell operating under ultrahigh vacuum in the high temperature–pressure cell at 700 °C at 10 atm. Then, the gases hydrogen and carbon monoxide in a ratio of 1 were introduced in the system where the temperature was varied from 250 and 270 °C in a similar fashion as the FTS reaction.

Finally, samples were cooled to room temperature in a reaction gas atmosphere. After transferring back to the spectrometer chamber for analysis, the samples were analyzed without exposure to laboratory air.

## 3 Results and Discussion

### 3.1 Surface Properties

XPS analyses were carried out to evaluate the chemical state and the superficial composition of the fresh FC1 and FC2. Figures 1a and 2a illustrate the survey and high-resolution spectra of selected core levels. The XPS survey spectrum of FC1 (Figure 1a) corresponds to the states of the Fe 2*p*, O 2*p*, and C 1*s* core levels, besides the Fe LMM emission Auger lines. Some contaminants, represented by the lines of K 2*s* and Cl 2*p*, arise from the mesoporous polystyrene carbon carrier that appears also in the XPS survey spectra.

**Figure 1:**
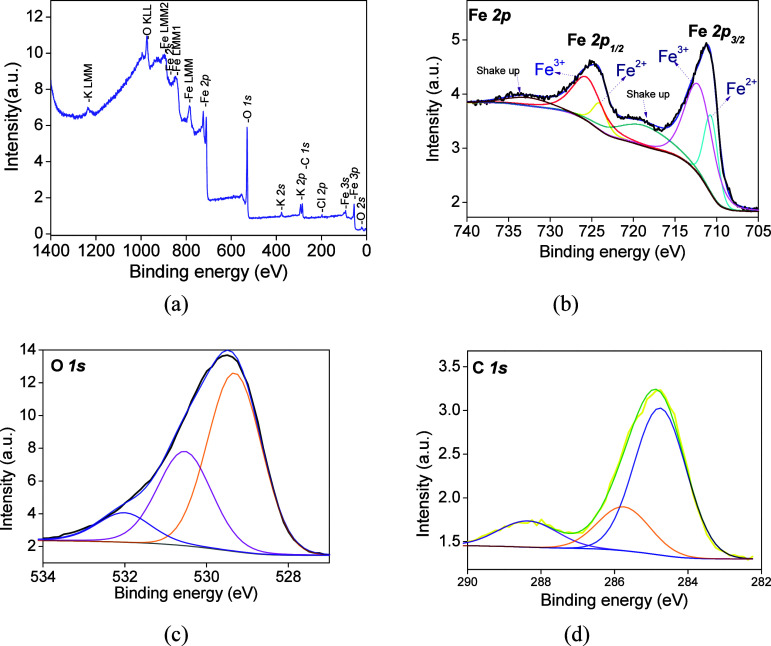
Photoelectron spectra of the FC1 sample: (a) survey spectrum, (b) Fe 2*p*, (c) C 1*s*, and (d) O 1*s* core levels.

**Figure 2:**
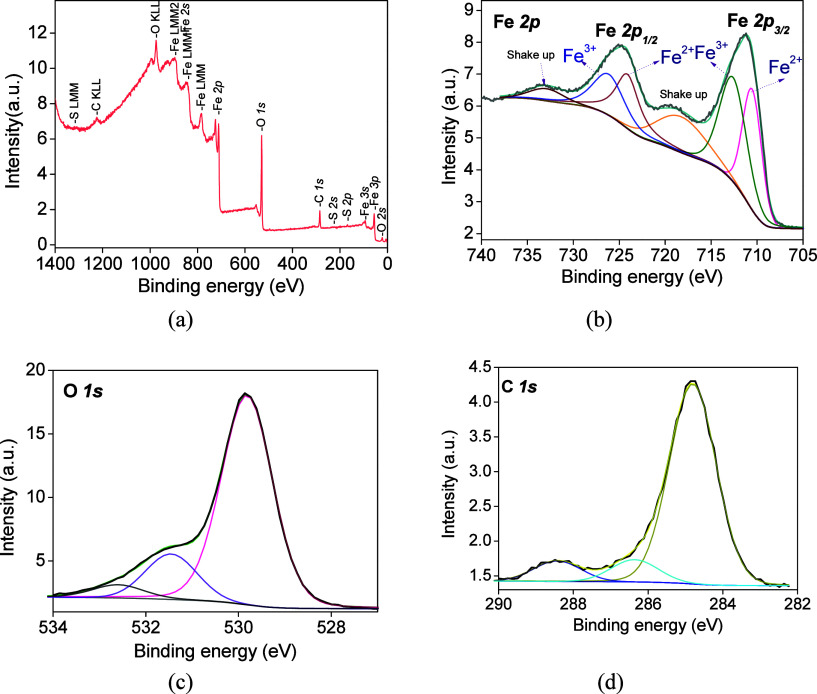
Photoelectron spectra of the FC2 sample: (a) survey spectrum, (b) Fe 2*p*, (c) C 1*s*, and (d) O 1*s* core levels.

The typical XPS spectrum of the Fe 2*p* core levels for FC1 (Figure 1b) appears as a high-intensity deconvoluted peak at 710.2–712.8 eV and a small one at 724.5–726.4 eV, which can be assigned to the Fe 2*p* *_3/2_* and Fe 2*p*_*1/2*_ contributions, respectively. These two spectral lines are associated with the difference in *j–j* spin–orbit coupling and the degeneracy of states that make the intensity of the Fe 2*p_3/2_* peak higher than the Fe 2*p_1/2_* counterpart.^19–21^ Likely, the dominant chemical state of iron in the sample is found to be Fe^3+^ as in the case of the α-Fe_2_O_3_ phase, which is previously observed by the solid characterizations.^3^ The appearance of the charge transfer satellite peaks at 718.1 and 732.2 eV is likely ascribable to the ejection of the core 2*p* photoelectron. This is in reasonable agreement with the electron transition from an Fe 3*d* orbital to the empty Fe 4*s* orbital in Fe^3+^ species from Fe_2_O_3_.^23–25^ Also, at 55.3 eV, the presence of Fe^3+^ species as the majority specie is confirmed (Table 1). It is worth noticing that the presence of either Fe^3+^ or Fe^2+^ species as γ-Fe_2_O_3_ is detected through the binding energy values, in similar peaks and satellite position ranges as those of literature reports.^24,25^ Consistently, the existence of Fe^2+^ is confirmed through the Fe 3*p* peaks in the survey spectra of all samples with binding energy close to 53.5 eV (Table 1), and in good agreement with the findings.^24,25^ The origin of the Fe^2+^ entities is the partial reduction of Fe^3+^ species by the CO released from the carbon matrix itself during the calcination.

**Table 1:** Binding energy values given in eV for Fe 2*p_3/2_*, Fe 3*p*, O 1*s*, C 2*p*, K 2*p*, K 2*s* and Cl 1*s* core levels for the samples.^a^

Sample	Fe 2*p_3/2_*	Fe 3*p*	O 1*s*	C 1*s*	K 2*p*	K 2*s*	Cl 2*p*	C/Fe	Fe^3+^/Fe^2+^
FC1		55.4	529.3(57)	284.8(66)				0.72	2.4
	710.2	53.4	530.5(32)	285.7(20)	292.6	377.1	197.8		
	724.5		532.0 (10)	288.4(14)			199.4		
FC2			529.8(79)	284.8(82)				0.93	2.2
	710.7	55.3	531.4(16)	286.4(9)	292.6	377.2			
	724.8	53.2	532.6 (5)	288.4(9)					

a The carbon-to-iron and Fe^2+^/Fe^3+^ atomic ratios are taken from XPS spectra. The numbers in parentheses are the percentage of the areas corresponding to each peak.

This observation is consistent with different iron species existing in the Fe^2+^ state as a result of the decrease in the coordination of Fe^3+^ ions at the surface.^8,25–27^ Accordingly, the Fe^3+^/Fe^2+^ ratios illustrate that the iron surface species existed in more than one oxidation state (Table 1), showing the abundance of surface Fe^3+^ species for all samples.

Noticeably, the two samples depict distinctive features concerning only the Fe 2*p* core levels (Figure 2b). Overall surface features of FC2 have very similar lines for C 1s and O 1s core levels (Figure 2c,d). Compared with the binding energies of the FC1 and FC2 samples, that of deconvoluted Fe 2*p* in the FC2 shifts to lower values than the others (Table 1). At present, it is difficult to distinguish between these possible contributions, that is to say, whether either the carbon carrier plays a strong interaction with both hematite and maghemite phases, forming other different phases, or the carrier promotes a role in reducing Fe^3+^ to Fe^2+^ species.

Furthermore, Raman and Mössbauer measurements will give insights into elucidating the influence of the metal species on the stabilization of iron oxide mixed phases.

In all cases, the data can readily be explained considering that the Fe species are in major concentrations on the external surface of the former solids, i.e., C/Fe atomic ratio of 0.72–0.93 (Table 1).

When considering the α-Fe_2_O_3_, γ-Fe_2_O_3_, and Fe_3_O_4_ iron oxide phases, the limited resolution in the XPS spectra does not allow distinguishing between the aforesaid phases in the samples. This is further confirmed by Mössbauer and Raman measurements.

XPS spectra of O 1*s* core levels for FC1 (Figure 1c) depict three fitted curves associated with different oxygen atoms coordinated to the iron species. The binding energy of the first component has a maximum at 529.5 ± 0.3 eV arising from the O^2–^ lattice oxygen in Fe_2_O_3_, and that for the other is in the range of 530.1–531.8 eV, belonging most likely to OH^–^ chemisorbed oxygen species or defect-oxide species on the solid surface.^3,24^ This peak is attributable to oxygen vacancies as in the case of Fe-based oxides.^23–25^ The third peak at 533.1 ± 0.3 eV matches well with the reported results for the presence of adsorbed water.^24,27^ Moreover, the relative abundance of these kinds of oxygen species suggests that the solids contain fewer hydroxyl-like and defect-oxide species than the lattice oxygen ions. It is generally accepted that the mixed metal oxides have characteristic surface lattice O^2–^ species, as a result of the intensive dehydroxylation of the oxide surfaces and desorption of oxygen-containing species formed during the high temperature of calcination such as at 700 °C. In addition, FC1 has an O_530_/O_531_ ratio of 2.1 while only a marginal effect of the aforesaid ratio is observed for FC2. Such an effect can be read as the local electron density distributions in the close vicinity of iron surface atoms that differ significantly with the FC2 sample, contributing the majority with oxygen defects. Moreover, the surface lattice O^2–^ species are bonded to the neighboring metal atoms; it is possible to ascertain that the samples possess vacant defects in similar positions of those of the lattice oxygen, according to the XPS results.

The C 1*s* core-level spectra of all samples (Figures 1d and 2d) are split into three components at 284.7, 285.7, and 288.3 eV. The first peak at 284.7–284.9 eV is attributed to adventitious carbon as a major component, whereas the peak positioned at 285.7–286.7 eV is mostly due to the C–OH/C–O–C bonds.^22,24^ The peak at 288.3 eV is associated with C=O bonds forming carbonyl and carboxyl species.^24^ It is clear that the origin of all these carbon species is believed to be from their chemisorption on the Fe sites preferentially during the synthesis or calcination. In addition, the K 2*p* , K 2*s* and Cl 2*p* core levels arise from the impurities of the mesoporous carbon support. The surface C/Fe atomic ratios are close to the bulk. In addition, iron carbides are not detected by XPS of the fresh solids.

### 3.2 Mössbauer Spectroscopy

Mössbauer spectroscopy provides identification of all iron species of the solids and their particles. As carburization of iron oxides hardly occurs on larger Fe particles, Mössbauer spectroscopy could predict the particle size to give insights on the carbide phase formation during *in situ* XPS analyses.

Figure 3a illustrates Mössbauer spectra taken at room temperature . FC1 shows sextet assymmetrical lines with Mössbauer parameters of δ = 0.36 mm s^–1^, Δ = −0.20 mm s^–1^, BHF = 51 T, and area = 100%. This is assignable to Fe^3+^ species from α-Fe_2_O_3_, and the negative values of the quadrupole splitting suggest a weak ferromagnetic feature typical of the hematite phase.^3,28^ This agrees very well with XPS results, which means that the Fe^3+^ entities on the surface of FC1 arise from hematite. It is worth noting that the hematite (α-Fe_2_O_3)_ has the corundum-type structure with a rhombohedral symmetry *R*3̅*c* space group. The trivalent Fe cations can occupy either tetrahedral and octahedral sites.^3,24^

**Figure 3:**
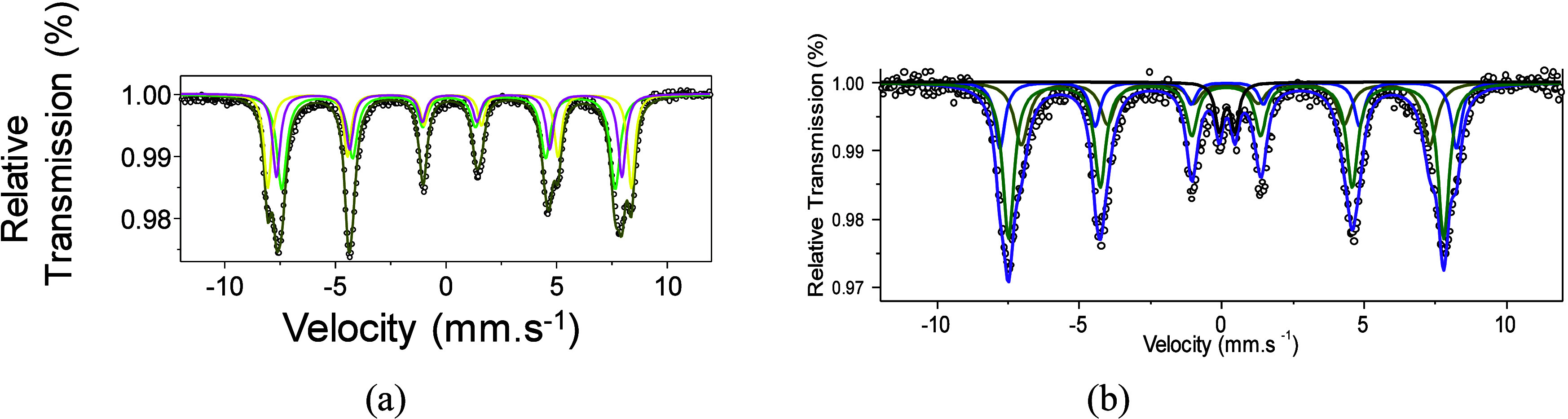
Mössbauer spectra of the catalysts taken at 23 °C for (a) FC1 and (b) FC2.

The Mössbauer spectrum of FC2 (Figure 3b) is fitted in two sextets with a hyperfine magnetic field around 51 T and isomer shifts of 0.31–0.37 mm·s^–1^ (Table 2). Such a feature is typical of the crystalline lattice site for Fe^3+^ in α-Fe_2_O_3_ weakly ferromagnetic having relatively small particle sizes or even γ-Fe_2_O_3_.^3,28,29^

**Table 2:** Estimated Mössbauer parameters obtained from the spectra at room temperature for selected samples^a^

Aample sites	Isomeric shift (mm·s^–1^)	Quadrupole splitting (mm·s^–1^)	Hyperfine magnetic field (T)	Area (%)
FC1 S1	0.36	–0.20	51	100
FC2 S1	0.31	–0.03	51	70
S2	0.37	0.0	24	
doublet	0.28	–0.01	50	30

a The symbols S1 and S2 represent sextets 1 and 2, respectively.

Both γ-Fe_2_O_3_ or Fe_3_O_4_ phases crystallize in a cubic symmetry belonging to the *Fd*3̅*m* space group of a spinel-type structure, as vastly documented.^25,27,30^ In such a structure, Fe cations located either octahedral (16d) or tetrahedral (8a) sites of the cubic spinel. Particularly, the inverse spinel structure adopted by magnetite and maghemite has octahedral sites occupied by a fraction or the totality of Fe^2+^ cations while the Fe^3+^ ions occupy octahedral and tetrahedral sites.^27^ All of these above observations manifest that magnetite and maghemite have a strong Zeeman splitting at ambient temperature owing to their ferromagnetic order.^31^ Taking into account that the Mössbauer features of Fe^2+^ cations are hardly distinguishable in maghemite, the hyperfine parameters are difficult to resolve.

The first sextet is associated with the Fe^3+^ ions on octahedral sites, and quadrupole splitting of −0.03 mm·s^–1^ correlates well with the hematite phase.^32^ The second sextuplet possessing a high isomeric shift of ca. 0.37 mm·s^–1^ seems to be from Fe^2+^ ions in tetrahedral sites. In that case, the second sextuplet may be attributed to the large oxidized maghemite particles. The S1 and S2 sextets represent 70% of the relative area, which is attributed to be from bigger particles, in line with the findings of refs 31,32 . A slight broad central doublet with isomer shifts of 0.28 mms^–1^ in the spectrum of FC2 can be due to the small particles exhibiting a fast superparamagnetic relaxation, as found elsewhere.^31^ These results assign the super exchange interactions between magnetic ions of the sites, confirming the presence of either hematite or maghemite in the sample. Moreover, the Mössbauer spectrum of FC2 depicts a main intense sextet and a broader centered doublet (Figure 3b). The doublet possessing 30% of the relative area suggests a rapid magnetic relaxation of Fe^2+^ ions most probably from maghemite and magnetite contributions. Moreover, the existence of the α-Fe_2_O_3_ and γ-Fe_2_O_3_ phases observed by Mössbauer spectroscopy is confirmed by the X-ray diffraction results.^3^

### 3.3 Raman Spectroscopy

Raman spectroscopy is a sensitive tool to metal–oxygen bond vibrations and lattice defects. Therefore, it constitutes a powerful tool to analyze the metal–oxygen interactions in oxides. In addition, the active phonon modes of the iron oxide main phases such as α-Fe_2_O_3_, γ-Fe_2_O_3_, and Fe_3_O_4_ can be clearly certified using Raman measurements.^27,33,34^ The Raman spectra of the samples are shown in Figure 4.

**Figure 4:**
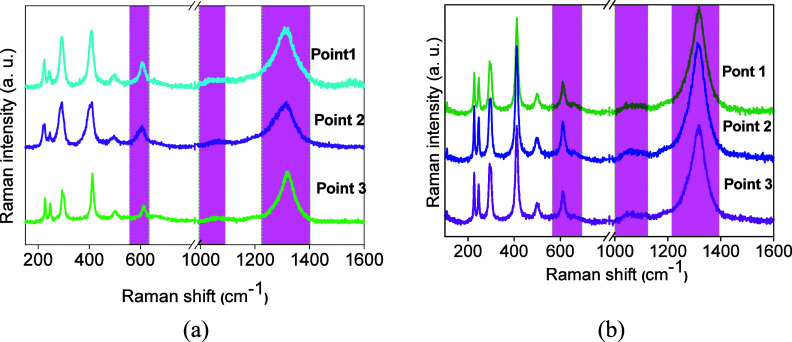
Raman spectra of the catalysts obtained at distinct points of the samples: (a) FC2 and (b) FC1.

The Raman spectrum of FC2 (Figure 4a) exhibits low-intensity mode vibrations at 221, 229, 293, 407, 495, and 603 cm^–1^ and a shoulder at 665 cm^–1^, in addition to an intense band centered at 1314 cm^–1^. According to the findings,^33,34^ hematite is an iron sesquioxide with a corundum-type lattice consisting of a Fe^3+^O_6_ octahedra, which crystallizes in a rhombohedral structure with a *R3c* (D^6^_3d_) space group. The group theoretical calculation for α-Fe_2_O_3_ revels seven Raman-allowed optical phonon lines owing to the Brillouin zone center.^34^ Theoretically, there are five internal modes, e.g., 2A_1g_ + 3E_g_ associated with the movement within a single FeO_6_ octahedral unit and two external E_g_ modes ascribable to the rotations and translations for α-Fe_2_O_3_.^24,27,33^

The Raman modes observed at low frequencies for FC2 appear at 228 (A_1g_), 247 (E_g_), 293 (E_g_), 410 (E_g_), 498 (A_1g_), and 613 (E_g_) cm^–1^ owing to the transverse optical (TO) contributions.^34,35^ Assignment of the obtained phonon lines is in accordance with the literature reports as a signature for α-Fe_2_O_3_ besides γ-Fe_2_O_3_ in FC2 spectra. It is important to note that a shoulder at around 661 cm^–1^ (highlighted in the pink square) can be due to the forbidden E*u* mode arising from the longitudinal optical (LO) contributions.^27,33^ Moreover, the origin of the intense band at 1310 cm^–1^ (highlighted in the pink square), the so-called 2LO longitudinal optical phonon, is associated with two-magnon scattering caused by the interaction of two magnons created on close antiparallel spin sites.^33–35^ There is a band at 1050 cm^–1^ rather broad than the other, whose position suggests that it is related to oxygen vacancy defects. It seems that these oxygen vacancies are present in the three points investigated of FC2 (Figure 4a).

Of importance, Raman spectra exhibit similar features in terms of position, when varying the points on the solid surface of the FC1 sample (Figure 4b). It resulted in the observed homogeneity of the surface of both solids. Nevertheless, the band at 1310 cm^–1^ for FC2 (Figure 4b, highlighted in pink square) shows a remarkable bordering in two points probably due to the poor scattering response. Consistent with XPS analyses, the surface of the samples is rich in Fe^3+^ species arising from hematite.

No significant differences of the Raman spectrum of FC1 (Figure 4b) are observed in terms of position and intensities, when varying the laser surface point, indicating the homogeneity of both surfaces. Mössbauer and Raman measurements evidenced that α-Fe_2_O_3_ with a rhombohedral structure is present in both solids.

### 3.4 Catalytic Performance in FTS Reaction

Catalytic performances of the solids in FTS synthesis are shown in Figure 5. From a first comparison among distinct temperatures (Figure 5a,b), there is a nonlinear temperature effect on the FTS performance on different catalysts. The solids are actives with conversion varying from 32 to 51% and a productivity of 1 × 10^–5^ mol_CO_·g^–1^·s^–1^, depending on the pressure and temperature conditions applied. Moreover, CO conversion does not increase proportionally with increasing temperature from 240 to 270 °C over all solids.

**Figure 5:**
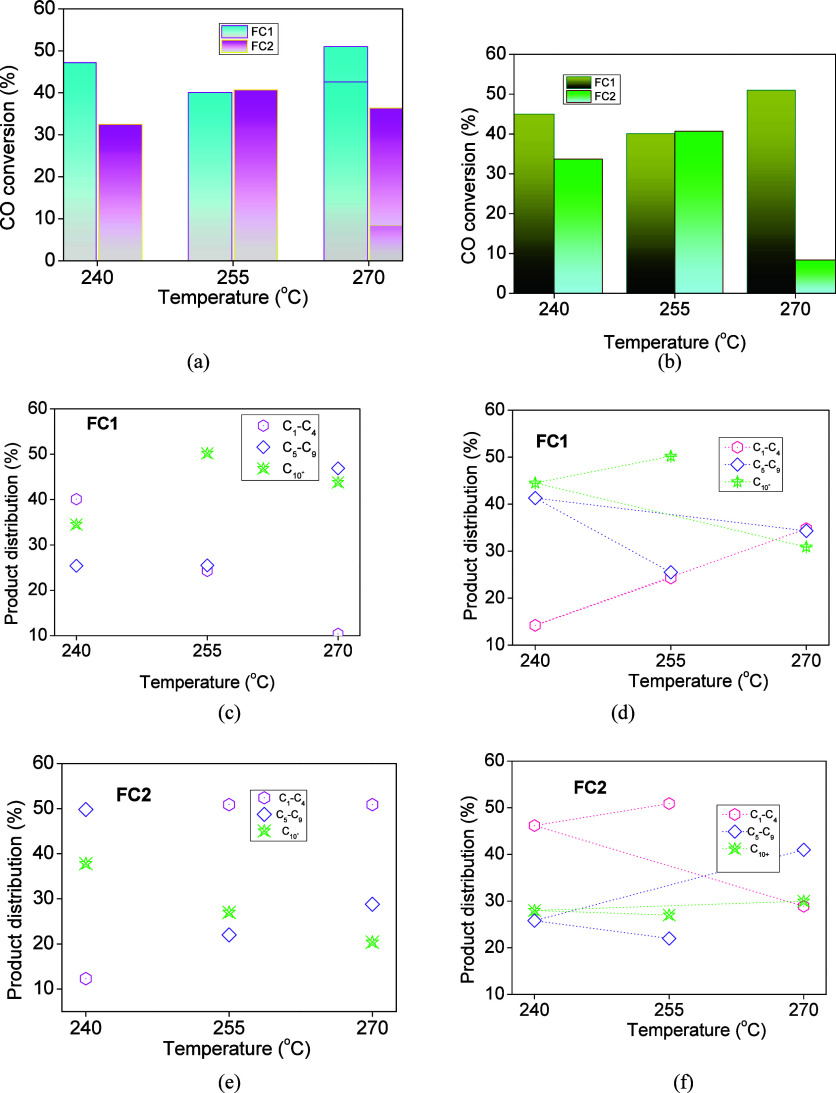
Catalytic evaluation in FTS synthesis: CO conversion in function of the temperature at (a) 20 bar and (b) 30 bar. Product distribution of (b) FC1 and (c) FC2 catalysts as a function of the temperature at 20 atm and CO-to-H_2_ ratio of 1 after 6 h of FTS reaction. Product distribution of (d) FC1 and (e) FC2 catalysts in function of the temperature at 30 atm after 6 h of FTS reaction.

Particularly, CO conversions for FC1 are found in the range of 47–51% (Figure 5a) upon increasing the temperature from 240 to 270 °C at 20 atm. As revealed by the Raman, Mössbauer, and XPS results, FC1 has mainly the α-Fe_2_O_3_ phase alone. When the latter rhombohedral phase is reduced, it is fully transformed into well-dispersed Fe_3_O_4_ nanoparticles on the mesoporous carbon support, as further demonstrated by *in situ* XPS analyses. Such spinel phase is accessible to CO_2_ and H_2_ and responsible for the catalytic performance in FTS reaction.^3^

Furthermore, similar trends are observed for FC2 with CO conversions reaching about 32.5–40.7% for temperature increments between 240 and 270 °C. Furthermore, the octahedral Fe^2+^ and Fe^3+^ ions located in the defective Fe_3_O_4_ play an important role during high-temperature WGS and FTS reactions.^36–38^ Accordingly, hydrogen involvement during CO dissociation promotes a redox reaction on the defective Fe_3_O_4_ sites possessing surface oxygen vacancies, with the latter being filled by CO to form surface carbonate intermediates. Concomitantly, water dissociation seems to be required on such oxidation of Fe^2+^ to Fe^3+^ to release hydrogen, whereas the oxidized Fe sites seem to be subsequently reduced by CO producing CO_2_ to complete the cycle.^38,39^ Such defect sites observed in FC1 by XPS may be expected to improve the CO conversions to a certain extent. Despite α-Fe_2_O_3_, the predominant phase with a minor amount of maghemite phase, FC2, is completely converted into magnetite during activation for FTS.

There are proposed reasons for the well-documented distinct reaction regimes for the FTS reaction governing diffusional steps at high temperatures. This indeed favors the occurrence of CO hydrogenation by WGS reaction (eq 4), as found elsewhere.^39^ Based on the fact that the Fe^3+^ and Fe^2+^ ions are present in the magnetite phase, the performance of FC1 and FC2 could be explained considering that the magnetite is the active phase for the WGS reaction. Therefore, it could be deduced that the catalytic activity of both solids toward FTS reaction, i.e., the extent of the reaction temperature increments effects on the CO conversions, is not greatly remarkable due to WGS reaction occurrence. Another possible reason is that the carbide formation such as Fe_*x*_C, in turn, may affect the FTS performance, despite the fact that the water formation arising from WGS results in the oxidation of the carbides concomitantly reoxidizing Fe^2+^ and Fe^3+^ sites from magnetite. This can enhance the WGS reaction, as confirmed by *in situ* XPS analyses.

Figure 5b illustrates the influence of the syngas pressure increment to 30 atm. It is expected that FTS performance would be enhanced up to a partial pressure of about 11 atm.^40^ Opposite trends are observed when the syngas pressure is increased for 30 atm while other process conditions are kept constant. FC2 is evaluated with only a slightly higher CO conversion of ca. 8% at 270 °C at 30 atm, while temperature as low as 255 °C favors CO conversions. The level of CO conversions rises almost linearly with syngas pressure of 30 atm over FC1, despite a lower reaction temperature of 240 °C.

Figure 5c–f shows the product distribution of the solids as a function of the temperature. The C_1_–C_4_ constituents are gaseous hydrocarbons, e.g., methane and C_2_–C_4_, while C_5_–C_8_ represents the gasoline fraction and C_10+_ compounds represent very long hydrocarbon chain fractions from kerosene with some of them from hydrocracking the latter into the diesel fraction.^41^ Moreover, the Anderson–Schulz–Flory distribution of the products with respect to carbon number can be determined by the chain-growth probability, which is considered independent of chain length.^42,43^ The distribution of the product analysis for FC1 (Figure 5c) illustrates the formation of ca. 40% C_1_–C_4_ products as a consequence of the hydrogenation side reactions to obtain either methane or carbon dioxide, short-chain products, and light olefins at 20 atm at 240 °C. At the same conditions, product distribution toward long-chain carbons such as C_10_^+^ within 35% is observed whereas C_5_–C_8_ production is marginally affected below to 255 °C. In contrast, there appears to be a preference over FC1 for C_5_–C_8_ and C_10+_ products compared to the C_1_–C_4_ ones at 270 °C. Accordingly, the CO conversion increment at high temperatures gives a decrement of light olefins at the expense of heavier product formation.^42,43^ This result can be explained considering that in the case of C_5_^+^ fractions, the water formation may result in the inhibition of hydrogenation reactions by competitive adsorption, which decrease the C_1_–C_4_ fraction with further olefin readsorption and growth at low space velocity.^43^ As the same active sites are present in both catalysts during activation, no differences between them can be observed.

When the product distribution is analyzed at 30 atm, distinct behaviors are observed. At 240 °C, FC1 shows the highest C_5_–C_8_ and C_10+_ production (Figure 5d), as observed under 20 atm, while the opposite is true for FC2 achieving the lowest one (Figure 5e). Again, both have a similar magnetite active phase after the solids are fully reduced, but a significant type of active carbide phase often formed during CO carbidization is thought to be the main reason for decoration of Fe particles with such species possibly affecting the product distribution over FC2 (Figure 5e,f). This is confirmed by *in situ* XPS analyses. Apparently, the carbidization does not depend on the catalyst type but its extension depends on the exposition on surface and particle sizes. The Mössbauer spectroscopy suggests that the particle sizes of FC2 are smaller than those of FC1 and, thus, CO carbidization is favored over FC2, as later confirmed by *in situ* XPS analyses.

By increasing the temperature from 240 to 270 °C, FC1 depicts achievable 30–34% of all fractions (Figure 5d), whereas the C5^+^ fraction around 41% was still detected predominantly at high temperatures over FC2 (Figure 5f). For this reason, both pressure and temperature do seem to affect the FTS performance with the distribution of the product dependence of the carbide formation appreciably.

Furthermore, *in situ* XPS investigations used a pressure of 10 atm at ∼240–270 °C, with a CO-to-H_2_ ratio of 1 during 1 h showing the evolution of the Fe 2*p*, O 1*s*, and C 1*s* core level spectra.

### 3.5 In Situ Operando XPS Investigations

Figure 6 depicts the XPS spectra of fresh, reduced, and recorded under relevant FTS reaction conditions for FC1. One has to mention that the original spectrum for the Fe 2*p* core level (Figure 6a, left panel in the bottom) exhibits spin–orbit doublets corresponding to the Fe 2*p_3/2_* and Fe 2*p_1/2_* contributions. As previously observed, the binding energies of 710.2 and 724.5 eV for the Fe 2*p_3/2_* core level assign the presence of Fe^3+^ from the α-Fe_2_O_3_ phase while the Fe^3+^ and Fe^2+^ species arising from γ-Fe_2_O_3_ are also shown. Moreover, the presence of shoulders as satellite peaks at binding energies of 732.3 and 718.2 eV for Fe2*p_1/2_* and Fe2*p_3/2_* contributions in the original FC1 confirms these assumptions.

**Figure 6:**
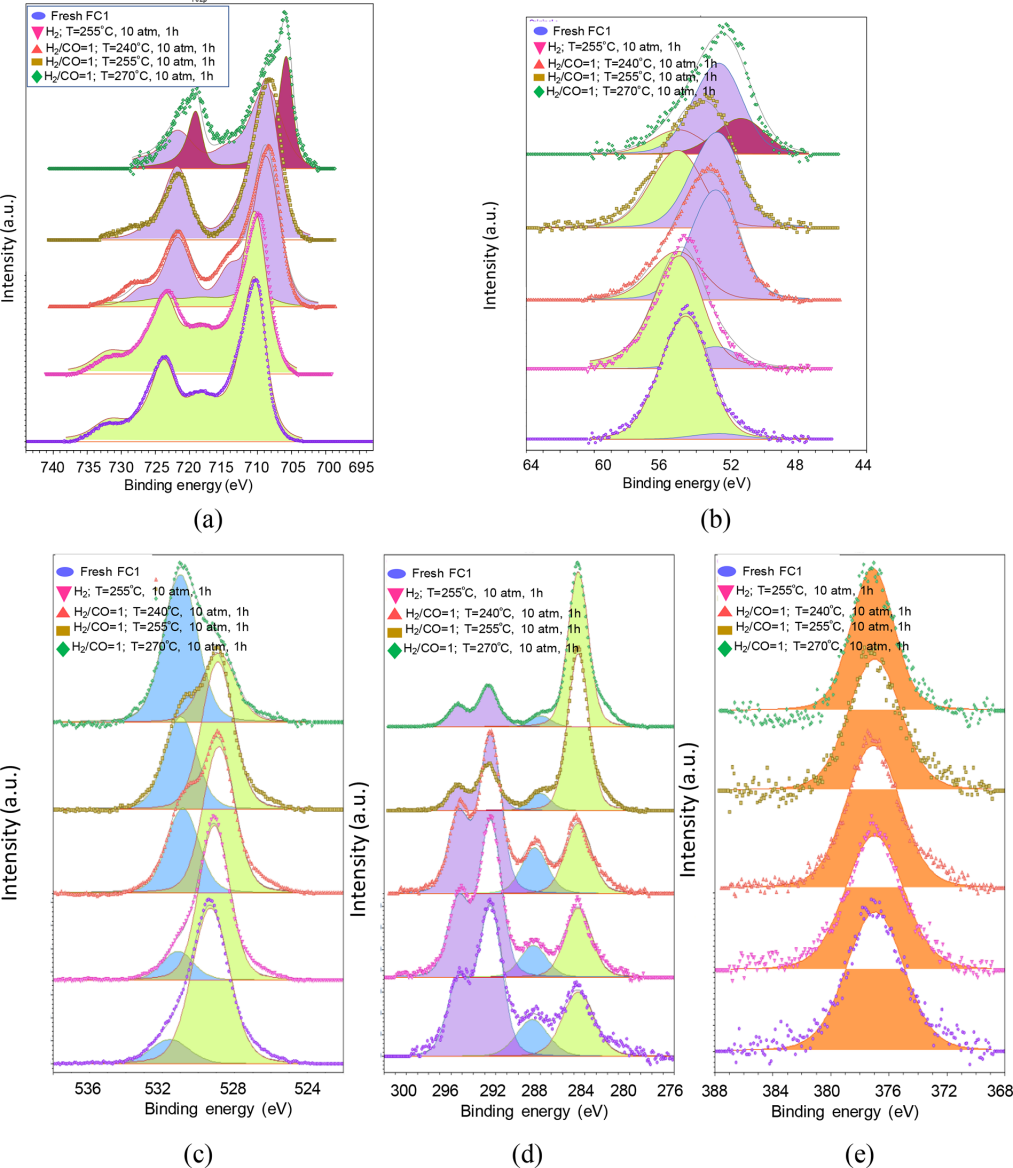
Evolution of (a) Fe 2*p*, (b) Fe 3*p*, (b) O 1*s*, (d) C 1*s*, and (e) K 2s core levels as a function of the binding energies for FC1. The spectra were recorded for the original sample (e.g., blue oval), reduced at 255 °C (e.g., pink inverted triangle), upon using a reaction mixture of CO and H_2_ with a ratio of 1 at 240 °C and 10 atm for 1 h (e.g., red triangle), using a CO-to-H_2_ ratio of 1 at 255 °C and 10 atm for 1 h (e.g., brown rectangle) and using a CO-to-H_2_ ratio of 1 at 270 °C and 10 atm for 1 h (e.g., green diamond).

Figure 6b in the right panel at the bottom displays the Fe 3p peaks for the original spectrum with the binding energy of 55.3 eV with a minor peak at 53.5 eV, suggesting the presence of the Fe^2+^ and Fe^2+^ contributions. A similar shape appears from the analysis of the Fe 2*p_3/2_* and Fe 2*p_1/2_* doublets acquired for the *in situ* reduced Fe 2*p* core at 255 °C for 1 h (Figure 6a, left panel in the bottom). In other words, a broadening of the Fe 2*p* peak and shifts toward lower binding energies is indicative of magnetite formation, as found elsewhere.^44^ This cannot exclude the reduction of Fe^3+^ species in the α-Fe_2_O_3_ and γ-Fe_2_O_3_ to Fe_3_O_4_ phase and its further reduction to metallic iron as the binding energy of Fe 2*p_3/2_* shifts to 708 eV while Fe 2*p_1/2_* moves to 718 eV, in agreement with the findings of refs 45,46 . This is consistent with the previous TPR results that exhibit the Fe_3_O_4_ reduction to FeO with its proceeding toward reduction to metallic iron, as shown in our previous work.^3^

Accordingly, the Fe 3*p* peak following exposure to hydrogen (Figure 6b, right panel at the bottom) indicates a change in the peak position, full width at half-maximum besides an asymmetric increment of the low binding energy peak at 53.0 eV along with a displacement of the 55 eV peak for higher binding energy, denoting the Fe^2+^ conversion to metallic iron.^45^

Through *in situ* XPS analyses at a pressure of 10 atm using a reaction mixture of CO-to-H_2_ ratio of 1 and a temperature of 240 °C, the phase evolution behavior of FC1 is illustrated. That is to say, deconvolution of the Fe 2*p* region reveals that the shape of Fe 2*p_3/2_* and Fe 2*p_1/2_* contributions hardly changed, despite the disappearance of the satellite near 719 eV, during the reaction under the given conditions. However, the deviations in peak positions and full width at half-maximum besides a more asymmetric Fe 2*p_3/2_* low binding energy peak accompanied by its downward shift provide compelling evidence for the formation of the iron carbides, which is consistent with the findings of refs 45–47 . This comes as a result of the surface–structure relationship showing that the Fe^3+^ and Fe^2+^ ions in the Fe_3_O_4_ phase are reduced to metallic iron along with iron carbides during the FTS reaction. As the carburization ability of metallic iron is higher than that of Fe_3_O_4_,^44^ the surface iron phases are concomitantly converted into iron carbides, i.e., Hägg carbide and cementite in addition to Fe_3_O_4_, both reaching a balance during FTS reaction along with the formation of hydrocarbon species on the solid surface. The experimental FTS reaction confirms these assumptions.

Importantly, the Fe 3*p* region (Figure 6b) has the intensity of the peaks positioned at 55 and 53 eV affected with the Fe entity present in the low binding energy peak as preferable for the structural change to form a mixture of iron partially reduced phases and their carbide analogues. In addition, it is not easy to ascertain the Fe components of the XPS spectra due to the uncertainties associated with the shape of the inelastic background of the Fe 2*p* region as well as the close proximity of the binding energies for the iron oxide phases.^47^

As the temperature goes up to 255 °C, the spectrum, recorded for the Fe 2*p* region at 710–724 eV, displays an obvious change as the intensity of the peaks gets higher followed by a shift toward low binding energies for Fe 2*p_3/2_* whereas the Fe 2p_1/2_ peak does not change so much. It could be indicative of the surface staying with the same species during the reaction and the surface carburization forming various iron carbide phases, mainly Hägg carbide (χ-Fe_5_C_2_), ε-Fe_2_C, and Fe_3_O_4_, and tentatively metallic iron phase, as latter shown. The Fe 3p peak clearly shows the presence of quite symmetric peaks with the one at 53 eV prevailing, in terms of intensity, revealing a greater formation of two iron carbides species. At 270 °C, the Fe 2*p* region exhibits the presence of four species between Fe 2*p_3/2_* and Fe 2*p_1/2_* lines (Figure 6a, left panel in the top) while gradually moving to higher binding energies as the reaction temperature increases. For the Fe 3*p* region (Figure 6b), a clear formation of three species at 270 °C is accompanied by their shift at lower binding energies and the intensity of the middle peak becoming higher than others. Such a feature is consistent with the favorable Fe_3_O_4_ spinel structure transformation into χ-Fe_5_C_2_ and ε-Fe_2_C along with Fe_3_O_4_ and metallic iron, when the temperature is increased up to 255 °C.

Figure 6c displays the corresponding O 1*s*core-level spectra for FC1. There are contributions of the O^2–^ lattice oxygen and defect-oxide species at 529.5 eV holding the dominant peak and 530.1–532.0 eV coming from the C–O/C=O/O–H entities, which is attributed to be from the mesoporous carbon support (Figure 6c, left panel, in the bottom). No clear differences are observed in the shape of the distorted O 1*s* of reduced FC1, although a development of the intensity of the signal at 530.1–532.0 eV is observed. It should be argued that the observed trends displayed in Figure 6 for the reduced samples are a result of all the α-Fe_2_O_3_ and γ-Fe_2_O_3_ transformations into a mixture of Fe_3_O_4_ and its partial reduction to metallic iron, as illustrated by the Fe 2*p* and 3*p* signals for the reduced FC1.

After syngas exposition at 240 °C, a very broad contribution of the peak at 531–532.0 eV appears at the expense of the slight shift of that at 529.5 eV toward low binding energies. Again, the iron carbide formation due to the adsorbed CO can be suggested along with the Fe_3_O_4_ and metallic iron particles. It is important to underline that due to OH adsorbed on the surface, the O 1*s* region could be a proof of favorable byproduct formation of *in situ* FTS reaction, as found elsewhere.^17^ At 255 °C, the significant width of the O 1*s* region signal at least twice as broad at 240 °C is explained by the coexistence of cementite θ-Fe_3_C and Hägg χ-Fe_5_C_2_ carbide species. These entities appear even though the lattice oxygen from the oxides or α-Fe phase is dominant. This is consistent with the result of the *in situ* XRD, EXAFS, and XAS available in the literature.^46,48^ It is worthy to note here that the temperature increment to 270 °C changes the O 1s core-level features with a detectable broadening of the peaks, suggesting that the fraction of iron carbide increased significantly covering the solid surface and any other Fe compound still present. Comparison of the reaction product distribution for FC1 reveals C_5_–C_8_ and C_10+_ heavy hydrocarbon products instead of light olefins, e.g., C_1_–C_4_ ones, whereas CO conversion varies over a wide range, i.e., 47–55% at 270 °C due to the concomitant CO hydrogenation by WGS and FTS reactions at high temperatures.

The C 1*s* core levels of fresh FC1 (Figure 6d, middle panel in the bottom) displays binding energies of 284.8, 285.7, and 288.4 eV, which is attributed to the adventitious carbon as the dominant component; C–OH/C–O–C, and C=O bonds form carbonyl and carboxyl species, respectively. At higher binding energies beyond 290 eV, a complex set of asymmetric peak and Π–Π* transition peaks is clearly visible, as found elsewhere.^49^ All of these contributions arise from the mesoporous carbon support. Accordingly, the O1*s* spectrum of the original FC1 (Figure 6c) shows the presence of C–O, lattice oxygen in oxides, and carboxylates at 529, 530, and 531 eV, respectively. In the C 1*s*core level of the *in situ* hydrogen reduced FC1, features similar to those of the original sample are observable.

When exposure of the syngas mixture at 240 °C is performed, the C 1*s* spectrum exhibits an increment in terms of intensity for the peak at 283.5 eV likely due to the adsorbed CO and C/CH chemisorbed on the surface, which results in the carbidic C–Fe bonds as iron carbides on the solid surface. Based on mechanistic considerations on Fe carbide surfaces during FTS,^50^ −^51,52^ the carbide surface forms a carbon vacant surface where CO dissociation and hydrogenation of the carbidic entity to CH_3_ monomers to HCO recover the carbon species through a dynamic behavior, in a similar way as the Mars–van Krevelen mechanism.^52,53^ In addition, the sp^2^-hybridized graphitic carbon is located in the C 1*s* region near 284.5 whereas the high binding energy at 285.5 eV assigns sp^3^-hybridized carbon such as amorphous carbon, both carbon species arising from the dissociation CO molecules.^17,52^ Upon increasing the temperature to 255 °C, the intensity of the peak at 283 eV gradually rises with the iron carbide predominance followed by a decrement in the area of graphitic and amorphous carbon at 285 eV, whereas a simultaneous drop in intensity of the asymmetric peak and Π–Π* transition peaks is observed. At high temperatures, the C 1*s* core level remains unchanged with a huge decrement of the peak intensities for graphitic and amorphous carbon, apart from a notable formation of iron carbide peaks. This result demonstrates that the iron carbide formation may possibly be thermodynamically favored over FC1, even though at FTS reaction, temperatures below 255 °C contribute to the surface carbon species owing to the limited diffusion of carbon from the surface into the Fe active phases.

The K 2s core levels are visible in Figure 6e (right panel) at 376 eV arising as impurities of the mesoporous carbon support, which remains unchanged during all the conditions evaluated. It is speculated that the addition of K in unprompted Fe catalysts for FTS suppresses the formation of light compounds such as CH_4_ and CO_2_ owing to the Fe–C bond strengthening provided by the increment in the electron density on Fe while weakening Fe–H and C–O bonds affecting the CH_*x*_ groups on the solid surface to obtain olefins and longer-chain hydrocarbon molecules.^54^ This can explain the product formation over FC1 compared to FC2 counterparts.

Another point worth mentioning is that the iron carbide (Fe_*x*_C_*y*_) structure consisted of carbon atoms located in available interstitial sites in a distorted hexagonal (hcp) lattice of zerovalent Fe atoms.^55^ Moreover, the crystal structure of the iron carbides is analyzed by the site occupation of the carbon atoms into trigonal prismatic carbides and octahedral carbides. For instance, the deformed hcp Fe sublattice is found in θ-Fe_3_C (*Pnma*), χ-Fe_5_C_2_(*C*2/*c*), and o-Fe_7_C_3_(*Pnma*), whereas the triangular prism interstitial sites are Fe_7_C_5_(*I2*), Fe_4_C_3_(*Cmcm*), Fe_5_C_4_(*C2*/*m*), Fe_6_C_5_(*Imm2*), and Fe_7_C_6_(*P63*/*m*).^55,56^ According to the findings, the octahedral carbides can be transformed into trigonal prismatic ones, depending on the temperature with martensitic shearing reaching up to 250 °C while that of χ-Fe_5_C_2_ above 250 °C and subsequently that of θ-Fe_3_C beyond 350 °C.^55^ Based on these findings, the θ-Fe_3_C, χ-Fe_5_C_2_, and Fe_7_C_3_ carbides are formed under the range of temperature and pressure experimentally applied in this work.

In an attempt to demonstrate whether the temperature of calcination plays an indispensable role in the FTS catalytic performance, the *in situ* XPS analyses of FC2 calcined at 700 °C is obtained (Figure 7). As FC2 is calcined at a higher temperature than FC1, it may provide on the surface of mesoporous carbon more functionalized groups that would result in a stronger interaction between the iron precursor and the carbon support with the consequent higher iron species dispersion, and thus, an improvement in the catalytic properties of FC2 is expected.

**Figure 7:**
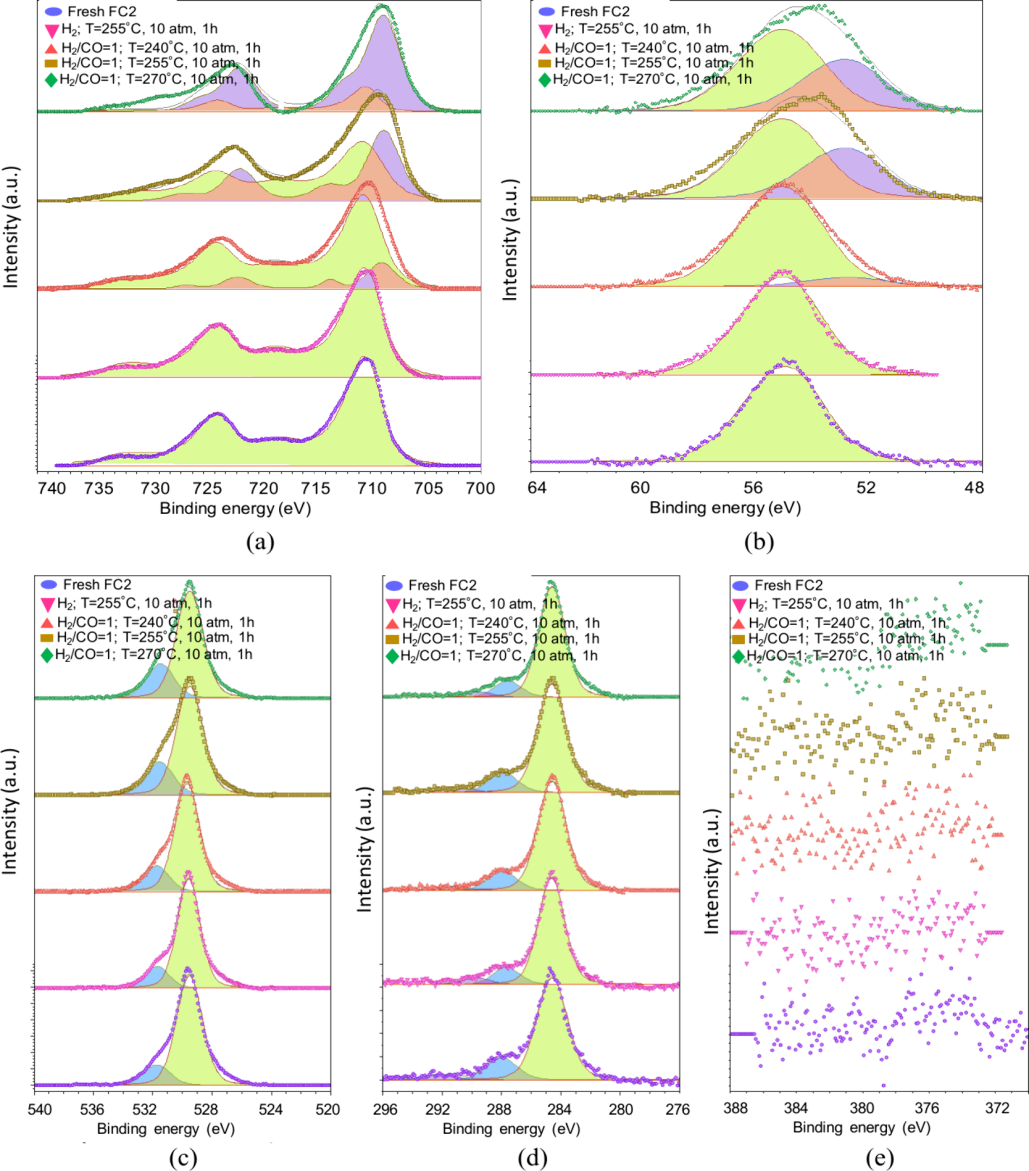
Evolution of (a) Fe 2*p*, (b) Fe 3*p*, (b) O 1*s*, (d) C 1*s*, and (e) K 2s core levels as a function of the binding energies for FC2. The spectra were recorded for the original sample (e.g. blue oval),, reduced at 255 °C (e.g.pink inverted triangle) upon using a reaction mixture of CO and H_2_ with a ratio of 1 at 240 °C and 10 atm for 1 h (e.g., red triangle), using a CO-to-H_2_ ratio of 1 at 255 °C and 10 atm for 1 h (e.g., brown rectangle) and using a CO-to-H_2_ ratio of 1 at 270 °C and 10 atm for 1 h (e.g., green diamond).

Despite the differences in the calcination temperatures, FC1 and FC2 hold similar Fe 2*p* core-level spectra (Figure 6a and Figure 7a panels, at the bottom). Concurrently, the Fe 3*p* core level for original and reduced FC2 (Figure 7b, middle panel, in the bottom) only has the peaks located at 55 eV with the absence of that at 53 eV, previously shown in the FC1 sample (Figure 6b right panel, in the bottom). This indicates that the Fe^3+^ species prevails in FC2 even after reduction. Particularly noticeable is the Fe 2*p* core-level shape of FC2 after syngas exposure at 240 °C, which is dominated by the well-developed Fe 2*p_3/2_* and 2*p_1/2_* peaks (Figure 7b, left panel). In addition, shifts in the peak positions followed by asymmetry of the Fe 2*p_3/2_* core-level peaks suggest the iron carbide formation. Accordingly, the Fe 3*p* peak slightly shifted to lower energy (Figure 7b, right panel), and the clear appearance of the peak at 53 eV assigns the formation of iron carbides. These results indicate that Fe_3_O_4_ is undergoing reduction on the proximal α-Fe sites in the carburization occurrence. Similar features are also observed for the Fe 2*p* spectrum at 255 °C regardless of the iron carbide formation. This underscores the idea that Fe 2*p_3/2_* shifts to the low binding energy region and increases the Fe 2*p_1/2_* peak intensity yielding iron carbide phases such as Hägg carbide χ-Fe_5_C_2_, ε-Fe_2_C, and Fe_3_O_4_. At temperatures above 255 °C (Figure 7a, left panel on the top), the Fe 2*p_3/2_* and Fe 2*p_1/2_* core levels indicate the same species as those of FC1 at 270 °C. The Fe 3*p* XPS spectrum (Figure[Fig fig7]b, right panel) gives further evidence of the iron carbides from the appearance of two high-intensity deconvoluted peaks at temperatures above 255 °C under exposure to the syngas. The findings state that ε-Fe_2_C is formed under carburization of the metallic iron at temperatures below 270 °C, but the instability of the latter phase provides the χ-Fe_5_C_2_ and θ-Fe_3_C formation.^55^ Thus, the obtained Fe 2*p* and Fe 3*p* results also confirmed the formation of such types of carbides as well as the iron oxide phases at high temperatures during FTS reaction.

Figure 7c (left panel) depicts the XPS spectra of the O 1s core level for FC2. Both original and reduced samples exhibit two major peaks at 529 and 531–532 eV (Figure 7c left panel, in the bottom), which corresponds to the O^2–^ anions in the lattice of α-Fe_2_O_3_, γ-Fe_2_O_3_, or Fe_3_O_4_ oxides and the C–O/C=O/O–H species from the support, as observed in FC1. Binding energy positions of the corresponding two peaks are shifted roughly toward lower-energy regions along with a higher intensity of the peak located at 531–532.0 eV upon using syngas at the temperature range 240–270 °C (Figure 7c left panel at the top), ensuring the iron carbide formation besides the water probably from the WGS reaction. This coincides with the observed O 1*s* core-level features of FC1.

The core-level C 1*s* spectrum for FC2 is shown in the middle panel of Figure 7d. Both original and reduced spectra (Figure 7d middle panel, in the bottom) reveal a deconvoluted doublet located at 284.5 and 288.4 eV, accounting for the C–OH/C–O–C and C=O bonds forming carbonyl and carboxyl species. At temperatures between 240 and 270 °C (Figure 7d middle panel, in the top), there is evidence that all of the peaks have the same shape with the high binding energy peak positioned at 283 eV while an enhanced intensity is perceptible similarly to FC1. Such shifts are attributed to a considerable intensification in C bonded to the iron on the surface of FC2 assigning the carbide formation.

Additionally, the K 2s core levels at different FC2 conditions (Figure 7e right panel, in the top) are featureless since K is not present in the support.

Considering the fact that the active sites of FC1 and FC2 are identified as the same, the composition of these samples differs only in terms of calcination temperature and the lack of K in the support formulation of the latter sample. Roughly, FC1 and FC2 have similar CO conversions owing to the Fe particle dispersion on the mesoporous carbon support. As a structure-sensitive reaction, the catalytic performance in the FTS reaction is associated with the nature of the active phase and its dispersion. Furthermore, *in situ* XPS analyses suggest that the product distribution of FC2 for long-chain hydrocarbon molecules and heavy olefins is a result of the stable χ-Fe_5_C_2_ and θ-Fe_3_C phases, which indeed suppressed coking covering the aforesaid iron carbide phases under the FTS conditions.

## 4 Conclusions

The surface sensitivity of mesoporous carbon-supported iron catalysts was investigated by *in situ* XPS analyses. Two Fe-based catalysts calcined at distinct conditions of temperature, *i.e*., 500 and 700 °C, exhibited quite different structural properties as proved by Raman and Mössbauer measurements. When reduced by hydrogen, both solids hold the same Fe_3_O_4_ active sites, roughly exhibiting similar CO conversion owing to the Fe particle dispersion and K presence on the mesoporous carbon support. The structure-sensitive FTS reaction activity was associated with the nature of the active phase and its dispersion. The surface–structure relationship investigated by *in situ* XPS analyses indicated that the α-Fe_2_O_3_ reduction to Fe_3_O_4_ is well dispersed on the mesoporous carbon support and its further reduction to metallic iron. The Fe 2*p*, O 1*s*, C 2*p*, and K2s core level evolution upon temperature increment up 255 °C revealed the surface carburization forming χ-Fe_5_C_2_ and θ-Fe_3_C iron carbide phases, along with Fe_3_O_4_ metallic iron phases. The product distribution displayed preferential long-chain hydrocarbon and heavy olefin formation as a consequence of the stable χ-Fe_5_C_2_ and θ-Fe_3_C phases, which may suppress coking of the aforesaid iron carbide phases under the FTS conditions in study.

## Data Availability

The authors have permission to share data under request.
